# Why do doctors in Norway choose general practice and remain there? A qualitative study about motivational experiences

**DOI:** 10.1080/02813432.2020.1753348

**Published:** 2020-05-12

**Authors:** Inga Marthe Gronseth, Kirsti Malterud, Stein Nilsen

**Affiliations:** a Research Unit for General Practice, NORCE Norwegian Research Centre, Bergen, Norway;; b Department of Global Public Health and Primary Care, University of Bergen, Bergen, Norway;; c The Research Unit and Section of General Practice, Department of Public Health, University of Copenhagen, Copenhagen, Denmark

**Keywords:** Career choice, motivation, general practice, life-work balance, medical education, qualitative research

## Abstract

**Objective:** To explore experiences motivating doctors to become and remain GPs.

**Design and contributors:** Qualitative analysis of written responses from an open-ended question about motivational experiences posted on an internet discussion list for GPs in Norway. Texts from 25 contributors were analysed with Systematic Text Condensation, supported by theories about calling as motivation.

**Results:** Analysis revealed numerous aspects of motivation to become and remain a general practitioner. Inspirations from early experiences and skilled role models had conveyed values and offered insight into a fascinating world of care, gratitude and respect. Close and continuous relationships with patients provided GPs with humbling experiences and learning moments. Contributors described how these encounters became rewarding sources of insight and mutual trust, improving interpersonal skills. Also, the extensive variety of tasks during the workday and the space for autonomy and independence was emphasised.

**Implications:** Understanding motivational experiences influencing GPs’ choice of medical career is necessary to develop strategies for recruitment and stability and contribute to prevention of burn-out and improper work-life balance. GPs’ professional identities and commitments should be recognized and developed in dialogues between authorities and GPs to enhance communication, improve the structural frames of work environment and thereby sustainable recruitment.

**Key points**
- GPs regard their choice of medical career as strongly influenced by motivational experiences in childhood, adolescence and as medical students
- Role models, diversity of work, feelings of being able to contribute and rewarding and continuous relationships with patients were mentioned to activate and maintain general practice commitment
- Knowledge about motivational influences, professional identities and commitment for GPs is crucial for medical education and dialogue to promote general practice as a career choice and prevent dangers of work overload and burnout

## Introduction

Primary care holds a strong position as the cornerstone of Norwegian healthcare. Since 2001 all Norwegians have had the right to choose a general practitioner (GP) as their regular doctor, and 99,3% of the population were enlisted in 2017 [1]. The national regular GP scheme rates among the most valued public services in the country [2]. In 2012, the Coordination Reform was launched to improve collaboration between primary and secondary healthcare [3]. After this reform, GPs have experienced a steady increase in responsibilities and number of tasks, thereby exceeding the 37.5 regular working hours in Norway by approximately 20 h per week [4]. A study among Danish GPs, where similar structural challenges are evident, showed large variations in job satisfaction [5]. One in four reported high job satisfaction and mental well-being, but half of them reported one or more symptoms of burnout. In Norway, statistics on GP shortage [6] suggest similar challenges. Job satisfaction remained stable and high for all medical specialities in Norway from 2010 to 2016, except for GPs for whom job satisfaction scores fell significantly [7].

Recently, Norway has seen a sharp increase in vacant GP positions, indicating an alarming crisis of recruitment and retention. Previous studies have identified several factors that influence the choice of a career in general practice such as early exposure to primary care, role models, variety, job stability, skill levels, income and work-life balance [8–12]. In contrast, little is known about GPs’ motivations for their career choice and how such factors influence the balance between professional commitment and burnout.

Wrzesnievski et al. [13] suggest that people relate to their work as either a job (focusing on financial rewards and necessity), career (focusing on advancement) or calling (focusing on enjoyment of fulfilling, socially useful work). Work as a *calling* has been defined as ‘work that is performed for its own sake, for the personal meaning and value associated with it’ [14] or as ‘work that a person perceives as his purpose in life’ [15]. We found these perspectives relevant to explore the current state of affairs. Sharing a strong enthusiasm for the profession, the authors have all experienced the recent challenges in general practice from different perspectives. We therefore decided to explore how motivational experiences reported by GPs in Norway contributed to their choice of a career in general practice and made them remain in the profession.

## Design, material and methods

We have conducted a qualitative study based on empirical data from an open-ended question posted on an internet discussion list for GPs in Norway.

### Data collection

Data were drawn from comments in a restricted Facebook group for GPs in Norway. This group is strictly moderated and open only to currently practicing GPs. As of spring 2018 the list included 3357 members, of whom approximately 50 participated regularly in discussions [16]. The age distribution among list members is younger than average for GPs in Norway, while gender is approximately equally distributed. In December 2017, the first author invited all members of the group to share experiences and reflections on becoming a GP. No questionnaire was presented but the following cues for open-ended answers were given: When did the contributors decide to become GPs, why did they become GPs and not gynaecologists, was this a conscious choice or did it just happen, were they inspired by someone or inspired by the profession itself, did they try a hospital-specialty first and does life as a GP meet your expectations? We encouraged them to give realistic contributions in a positive spirit. Although not part of the original question, contributions included substantial information on motivational factors for remaining in general practice. This information was therefore included in the final analysis. Informed consent to use the material for research was obtained via Messenger from 25 contributors (16 women and 9 men). None of the contributors were excluded from the study. Age was reported by 20 of them (median 44 years, range 33–69 years).

The contributions varied in size (range 34–1288 words, median 178 words) and contents. The majority included specific and thick descriptions [17] of experiences motivating the contributors to choose general practice as their medical field of work. When incoming responses terminated, we assessed information power in our convenience sample, considering study aim, sample specificity, established theory, quality of dialogue and analysis strategy [18]. Our study had a relatively narrow aim, and the sample shared specific experiences of becoming and remaining a GP. Theories on calling and job motivation supported analysis. The dialogue quality was strong – even when relying on one open-ended question, the contributors were articulate and provided diverse variations. Although cross-case analysis strategy requires sample size beyond a few individuals, we concluded that our sample size of 25 held appropriate information power for analysis.

### Analysis

We conducted analysis with systematic text condensation, a method for thematic cross-case analysis of qualitative data [19]. The analysis involved the following steps: (i) Read the material, gain an overview of data and elicit preliminary themes, (ii) develop code groups from preliminary themes, identify and code meaning units reflecting the contributors’ experiences of their choice of career and their jobs as GPs, (iii) establish subgroups exemplifying vital aspects of each code group, condensing the contents of each subgroup and identify illustrating quotes, and (iv) synthesize the condensates from each code group to a reconceptualised description of aspects of motivation for working as a GP. IMG and KM read the material and participated in analysis and interpretation. Code groups and subgroups were negotiated. Analysis was further elaborated by SN in the later steps of writing. Perspectives about calling supported our analysis as substantive theory offering a sharper focus for interpretation. Theory was used neither as template framework, predetermined categories nor as a model to be empirically tested [13,20–22].

## Results

Our analysis revealed numerous aspects of motivational experiences influencing doctors’ decision to become and remain GPs. Contributors described how inspirations from early experiences and skilled role models had conveyed values and offered insight into a fascinating world of care, gratitude and respect. Close and continuous relationships with patients provided GPs with humbling experiences and learning moments. They described how these encounters became rewarding sources of insight, mutual trust, improving interpersonal skills, and sometimes offering an opportunity to aid in instituting a major change in a patient’s life. Also, the extensive variety of tasks during the workday, and the space for autonomy and independence made some of the GPs label the specialty as ‘the best of all’. Below, we elaborate on these findings. Quotations are marked by the number assigned to the actual contributor.

### Skilled role models facilitated entrance into the profession by sharing knowledge and values of care, gratitude and respect and providing safe refuges for reflection

Many contributors described the impact of being personally and positively exposed to doctors and doctoring, often from an early age. Formative impressions could be instigated with a parent running a general practice in the same house, or in a village where the country doctor was often seen in action as an esteemed professional and community member. In such contexts, contributors described how values like care, gratitude, politeness and shared knowledge made an impression on a young person. Own patient-experiences at an early age were mentioned as influential background imprints.

Some contributors described how cultural ideals, such as the ‘serving the people’ slogan from the 1970s, had an impact upon their career choices. Attending a medical school where general practice was highly esteemed had stimulated their pursuits. Contributors explained how enthusiastic teachers had triggered a contagious curiosity by sharing the distinctive character of the discipline. They described how their fascination with home visits, sometimes in remote settings by ambulance boat, intimate encounters and unexpected medical events provided them with values and inspiration beyond learning. For some of the contributors, these weeks in general practice as medical students had contributed to a feeling of belonging, satisfaction and coping which had previously been lost in the university hospital culture. Some of them told about how they decided during the practice period that this would be their future home ground.

Experiences of inspiring role models were mentioned by several contributors. They had been allowed to observe how sincere listening, reflection, trust and respect could be enacted by well-informed and skilled supervisors. The seniors shared their tools of the trade with the juniors in a way that enabled them to construct their own tools. Role models who made an impact were described as wise and warm, recognizing the students and always taking the time for listening and reflection when needed. One of the male contributors said:

*“I grew up in a small community, with the local GP as the role model whom I had seen in action as a child suffering from earaches, and as a youngster with injuries from ski-jumping. During medical school in X, I was inspired by skilled lecturers [yy, zz and colleagues] who conveyed the distinctiveness of general practice. (…) During rural clinical training I had an exceptionally skilled supervisor. He and his colleagues, with whom we also shared on-call duties, introduced me to a varied profession where I would really be challenged and would never become fully trained.” (#23)*

Several contributors referred to how senior mentors during internship in general practice had facilitated the junior’s entrance to the discipline including proficiencies as well as patient interaction. One of them described how her first day of internship gave her a convincing feeling of coming home, while others expressed how this training period had unambiguously confirmed their attraction to become a GP. Training groups, which in Norway are mandatory for general practice speciality, were also described as a structure of interaction offering significant professional relations and experiences, sometimes with a seminal impact on identity and coping. A GP emphasized the significance of this safe refuge for reflection, especially during a period of particular vulnerability:

*“I was very lucky to secure a spot in a training group proficiently led by xx when I started my career in general practice. I do not think I would work in general practice today if it had not been for this group. Such safe havens for reflection are exceptionally useful, especially during a time when one is particularly vulnerable.” (#19)*

### GPs experienced personal and professional gain from close and confident relationships with patients

Many contributors emphasized the continuity of care and the opportunity to get to know people over long periods of time as a primary reason to choose general practice over other specialties. They described how deep relationships with patients, following them through different phases in life, was the main reason why they loved their job. The contributors wrote enthusiastically about how they had gotten to know individuals in the local community. They had experienced how stories were shared in close doctor–patient relationships, including thoughts and problems that patients have carried alone over some time. They mentioned this knowledge as an important key to recognize relationships between problems and symptoms that the GP would otherwise not have discovered. A contributor stated it this way:

*“The great encounters with patients, experiencing that I can help, having the possibility to work with patients and families over time, to discover relationships between problems that can only be identified by utilizing the time factor.” (#15)*

The trust and confidence that patients have in their GP, and the ways they expose themselves in consultations to show their most vulnerable and inner selves were described by several contributors as unique and rewarding features of general practice. Many of the contributors felt humbled and honoured by the trust that patients showed them, and some also described gratitude for being able to serve the patient and giving something back. This unique interaction between doctor and patients in general practice was exemplified in several accounts. Some contributors reported that patients valued having them as a doctor and often expressed respect, which again provided the GP with a sense of achievement and gratitude. A contributor said:

*“I love my job and I am humbled by being given the opportunity to get to know so many wonderful patients. They share their innermost feelings and thoughts. Imagine that they have been able to build such great trust in little me! Also, I feel incredibly grateful to have this opportunity to give something in return.” (#14)*

When describing what they had gained professionally or personally from working as a GP, contributors mentioned how they had learnt to listen more closely, to explore and elaborate and not only see symptoms but the whole individual. Some spoke of how they had improved interpersonal skills such as listening carefully, expressing trust and showing respect, and sometimes even gained the possibility to inspire a major change in a patient’s life. While emphasis in general was placed on positive experiences, contributors also described how they went through taxing learning moments. This was exemplified by a GP who had a difficult time coping with the patients’ despair when an important community establishment had to close, and many employees lost their jobs. A GP described how learning moments through his practice life had provided him with personal gain:

*“What I appreciate most in my work, after several decades as a GP, is that I hopefully have become humbler and developed great respect for mankind, especially those who have experienced great hardships in life. This insight is what I appreciate the most.” (# 9)*

### The diversity of tasks in general practice requires broad knowledge and skills and provides a professional environment with exciting workdays

Many contributors argued that it is in the field of general practice that one can really make a difference. Some expressed that the diversity of the tasks and the need to be knowledgeable about everything made them think of this specialty as the best of all. They mentioned variation, independence but also the skills needed to run a business. Several highlighted that general practice is wide-ranging and at the same time close to everyday life, exciting and rewarding, offering workdays where they could utilize all their capacities. They explained how the extensive range of challenges, from simple and trivial to important and complicated problems, ensured workdays that were never dull. One contributor emphasized that every day she felt able to contribute somehow, even if she sometimes fell short. Others explained how working in a profession where competence and skill are strongly valued is a privilege. They added that the experience of providing efficient and independent medical service elicited a feeling of well-being and a sense of coping that made them look forward to every workday. Many of them stated that gradually, they became more confident in their role as a GP while still being curious about how to further develop in the field. A GP reflected on this:

*“I started because it was close to everyday life, versatile, exciting and rewarding. Every day I felt like I was able to contribute in some way, even though I often fell short. The combination of the large and the small, the trivial, the simple and the complicated have provided rewarding professional challenges in a job that has never become boring. It is more important to know what one does not know than to believe that it is possible to know it all.” (#7)*

The contributors expressed ardent feelings towards the diversity of their workdays, encountering problems that sometimes could be easily fixed and other situations where merely being present was most important. They appreciated the fact that many problems were not exclusively biomedical. The surplus value of seeing patients of all ages and genders and the challenges of managing emergencies side by side with offering comfort, being able to adjust to different needs and navigate between different concerns, was also emphasized. A young contributor exemplified this point:

*“The patient needing drainage of 20 ml hydrops liquid reminded me of the delight of practical procedures in between social problems and life crises. (…) Supporting patients who struggle with their lives and coping is so rewarding, even when it sometimes is taxing and frustrating work.” (#12)*

## Discussion

Different experiences and reflections motivate young doctors to choose general practice and remain in the profession. Early exposure to and interaction with role models conveying important personal and professional values, rewarding relationships with patients through continuity of care and great variety of tasks throughout the workday were emphasized. Below, we discuss the strengths and limitations of the study design and the impact of these findings.

### Strengths and limitations

Contributors were recruited from a Facebook group, where members might be particularly passionate and motivated for their work. GPs from different geographical locations contributed, representing both genders, rural and urban practises and a varied range of ages and experience. As participants are self-selected, these are likely to be the most enthusiastic GPs who were also willing to share information on this subject. Observing the responses from other members, we noticed no specific indications that these contributors were outliers in the GP community. As we explore neither distributions nor prevalence, we therefore consider such a selection to be an asset for the information power of the sample, not a bias. Our intention was not to cover a complete and comprehensive presentation of any possible motivation, but to share experiences and reflections relevant for development of strategies to recruit and retain GPs, and we concluded that external validity was satisfactory [23].

Collecting data from social media is different from interviews, since there is no ongoing real time dialogue. Conditions for communication are changed, often more oral and informal. While posting a contribution on social media might have resulted in some idealization of the GP’s text, this effect would be less prominent than in a focus group situation [24], and probably diminished by the Facebook group being open only to GPs. Our study design may have triggered consecutive associations from one contributor to the next, in a similar way to a focus group discussion. It is also probable that the most active and verbally gifted members of the group were the most likely to participate, though these members might also be those best suited to convey these motivational experiences.

While the limited format of the answers and the natural tendency to neglect less prominent experiences could have influenced our results, we found varied, vivid and often detailed descriptions of the GP’s experiences describing and reflecting on several aspects of motivation. These contributions indicate that enough room was left for the contributors’ interpretations, even though some cues for open-ended answers were given. Since these stories are retrospective accounts of motivational factors and experiences, they were also modified through time. Though, as our purpose was to explore the GPs’ current reflections on these experiences and the relation to their work, we do not consider the retrospective aspect a disadvantage. These elements must however be taken into consideration when interpreting results, as to not compromise internal validity [23].

The researchers are well known among GPs in Norway due to active participation in the GP community over years, which may have contributed to trust and participation. In addition, our experiences as GPs and our own contribution to the study facilitated dialogical coproduction and may have bridged the natural gap between researcher and contributors. Since our personal engagement may have drawn our attention to motivational factors that corresponded with our own experiences and preconceptions, we assessed a potential effect of this through every step of analysis [25].

### What does our study add?

Previous studies have explored factors influencing the choice of working in general practice as well as attitudes and perceptions regarding a career as a GP [9–12,26]. Our study confirms the impact of early exposure to the specialty, work-life balance, variety and flexibility and continuity of care as being among the known determinants [8,9,12,26]. Previous studies have, however, mainly been conducted among medical students or doctors in their early years of training. Furthermore, there is limited research about the meaning and impact of motivational factors and expectations throughout a career in general practice.

Our study adds to existing knowledge by recognizing and interpreting how motivational experiences and the impact of these for work and perception of work influence GPs throughout their career. We also offer insight into how motivational factors evolve and are influenced by practice experiences, and how this in turn may contribute to a lasting work motivation.

### Understanding GPs’ work experience and motivation as calling

Our findings are expanded by theoretical perspectives describing work as respectively job, career or calling [13]. *Calling* as a personally fulfilling or socially significant engagement is consistent with our contributors’ accounts when they emphasize general practice as rewarding and appreciate the potential to make a difference for patients in a profession where competence, skills and interaction are greatly valued. These perspectives were chosen as substantive theory complementing our data, not as an a priori theory to be tested empirically. Interpreting the work experiences presented by the contributors as calling sharpened our analytic focus and made it easier to understand why doctors – even under the current strenuous conditions – were motivated to become and remain GPs. A sense of calling contributes to the individual’s identification with an occupation, and affords a conviction of the significance of his or her profession within society [21].

While calling was originally a concept rooted in religious traditions, a modern and secularized version of calling denotes work providing personal as well as social significance, offering self-actualization and personal passion [21]. A study of zookeepers demonstrates how an idiosyncratic wiring outside oneself can make the choice of career predetermined, reflecting a sense of destiny actually more coherent with the classical conceptualizations of calling than with modern, more self-focused conceptualizations [21]. We find elements of such an existential sense of destiny in our study, for example in the ‘feeling of coming home’ to general practice. Most contributions were, however, more consistent with a modern conceptualization of calling, with clear aspects of personal choice based on a foundation of influences from preclinical years and internships.

Development of calling can be regarded as an ongoing process, evaluating the purpose and meaningfulness of activities within a job and their contribution to the common good and welfare [22]. Such a process is noticeable in our contributors’ descriptions of their work. GPs of different age and levels in their career express that the meaning and purpose related to their work are derived from role models, cultural influences and individual experiences leading to strong emotions like pride, coping, and gratitude. Meaning and purpose are also related to the diversity within the workday, and many GPs describe job variety as a vital factor contributing to persistent job satisfaction. This is in line with previous perspectives from work psychology, where variety, drawing upon several skills, appears as an almost invariable source of meaningfulness for the individual [27].

Recognizing *calling as a process* could indicate how the structure of the Norwegian medical education can enhance pre-existing motivational foundations. General practice is an important element in medical school, and a six-month general practice internship is mandatory for young doctors in Norway. This program allows juniors to (self-) explore and try out several specialties including general practice. In our study, inspiring experiences with skilled mentors and patient interaction in many cases enhanced the preference for general practice or confirmed an already existing interest in this specialty.

### A double-edged sword?

Most stories in our sample reflect a strikingly positive attitude towards general practice and a high job satisfaction among contributors. Our study question specifically encouraged positive factors contributing to GP recruitment and stability, thereby focusing less on negative aspects. Recent developments with increasing workloads and failing recruitment to general practice could, however, indicate that a sense of duty and responsibility, as recognized from calling theory, can lead to conflicting feelings towards working as a GP. Calling can have a double-edged nature [21,22], being a source of meaning, identity and significance but at the same time associated with sacrifice of pay or comfort for the sake of others [22]. As such, calling can bear negative implications for the individual [21]. Such altruism, exceptional professional dedication and willingness to put in extra effort regardless of the circumstances has also been described in a recent study exploring Norwegian hospital doctors opinions on being a good doctor [28]. Still, the challenges of a broad-ranging medical discipline, the ability to provide care from the cradle to the grave and the sense of being part of a community are aspects of general practice that are greatly valued [10,29].

Individuals who approach work as a calling, despite negative implications, have reported greater work and life satisfaction and greater commitment to their profession. They also report greater use of problem focused coping, less stress depression and avoidance coping, and attribute greater importance to their careers [22]. The concept of calling could possibly enlighten why these GPs, despite a considerable workload and increasing demands from patients as well as central authorities, remain in their jobs and report high job satisfaction and positive attitudes towards their work.

## Implications for practice, education and policy

Understanding motivational factors that influence GPs’ choice of career and a decision to remain in the profession is necessary to develop recruitment strategies to increase influx and stability. Knowing the background and implications of calling as job motivation among GPs could point to more specific aspects available for intervention, such as time management, guidelines and structures to aid in prioritizing of tasks. Such measures might in turn contribute to prevention of burnout and improper balance between work and leisure among current GPs. The nature of calling suggests that when we interpret our findings in light of this theory, it may be implicitly expected by GPs that authorities share their strong sense of moral duty. If so, GPs’ professional identities and commitment need to be recognized and developed in dialogues between authorities and GPs. This then could possibly enhance communication and improve the structural frames of the work environment for GPs. Such strategies can thereby retain current GP’s and ensure increased and sustainable recruitment to general practice in coming years.
